# Six events that shaped antibody approvals in oncology

**DOI:** 10.3389/fimmu.2025.1533796

**Published:** 2025-02-10

**Authors:** Suman Paul, Shibin Zhou

**Affiliations:** ^1^ Department of Oncology, Johns Hopkins School of Medicine, Baltimore, MD, United States; ^2^ Ludwig Center for Cancer Genetics and Therapeutics, Sidney Kimmel Comprehensive Cancer Center, The Johns Hopkins University School of Medicine, Baltimore, MD, United States

**Keywords:** antibody, oncology, bispecific antibody (bsAb), antibody drug conjugate (ADC), immune checkpoint inhibitor

## Abstract

A little over twenty-five years ago, the European Medicines Agency (EMA) and the Food and Drug Administration (FDA) approved the chimeric antibody rituximab which fundamentally altered the landscape of anti-cancer drugs. While only a few antibodies were approved in the immediate years that followed the rituximab approval, the last decade saw a wave of antibody-drug approvals in the oncology arena. In the last three years, the EMA and FDA greenlighted eighteen antibodies, the majority of them designed in the formats of antibody-drug conjugates (ADC) and bispecific antibodies (BsAb). While the use of ADC and BsAb formats and the current rapid pace of approvals appear routine and almost inevitable, such progress was thought to be quite improbable in the early days of therapeutic antibody development. To understand how we arrived at the current state of antibody development in oncology, we focus on six monumental events that shaped antibody approvals over the last two and half decades. We examine the circumstances that led to the approval of rituximab and trastuzumab, the first successful antibodies for the treatment of hematologic and solid cancers. We detail the generation of the ADC and BsAb formats that dramatically augmented antibody-mediated precision cytotoxicity. Finally, we explore the development of ipilimumab, the first immune checkpoint-inhibiting antibody that activates the immune system to kill cancer cells, and the discovery that allowed the use of checkpoint inhibitors across all cancer types based on the presence of genetic markers. Revisiting these key events provides critical insights into the process of antibody development in oncology.

## Main

**Figure 1:**
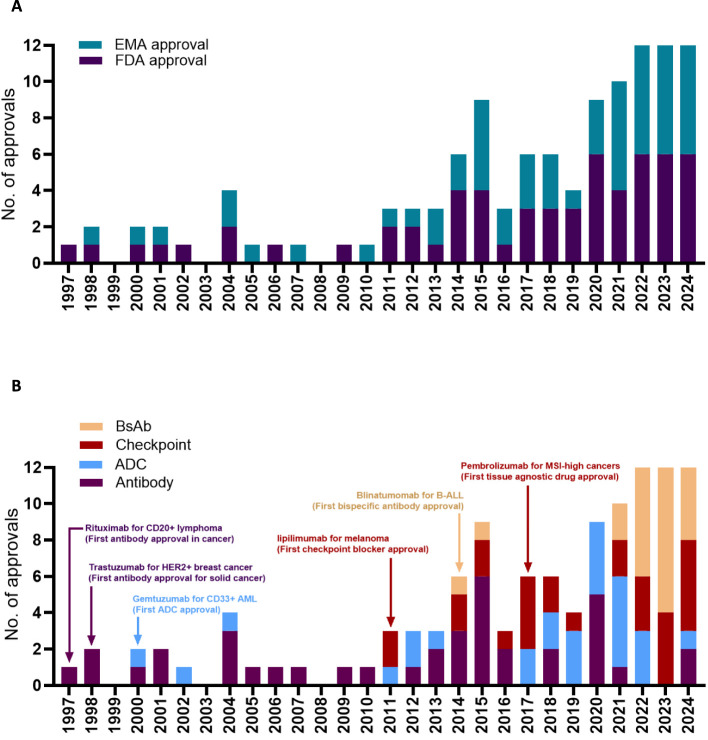
Timeline for antibody approval by the EMA or by the FDA **(A)**. Timeline for antibody approval based on the antibody format **(B)**. Edrecolomab targeting EpCAM was approved in EU for the treatment of colorectal cancer in 1995, but was subsequently withdrawn, and is not shown in the figure. Pembrolizumab was first approved by the FDA for the treatment of melanoma in 2014, followed by several successive approvals for different cancer types including the 2017 approval for MSI high or MMR deficient cancers. Data obtained from the Antibody Society.

Oncology now has the largest share of prescription drugs with steadily increasing revenues over the last decade (1). The explosive growth of cancer-targeting antibody drugs is due to a large patient population with significant unmet medical needs and ongoing innovations in antibody design led by academic laboratories and pharmaceutical companies. Antibody development in oncology driven by collaborative work from academia and industry has surpassed the growth of other cancer-targeting agents such as kinase inhibitors, hormonal drugs, cancer vaccines, and chemotherapies by a wide margin (1). What are the events in the last two decades led to the current state explosion of antibody-based therapies in oncology? Here we attempt to highlight six key regulatory approvals as case studies that fundamentally changed antibody-drug approvals by the FDA and EMA (**Figure 1A**).

### Rituximab, the first successful antibody for cancer therapy

In the 1990s, medical oncologists had a limited arsenal of cytotoxic drugs that they could offer to patients with cancers. Such cytotoxic agents were rarely curative and carried a long list of treatment-related adverse effects (2). Two seminal techniques developed in the 1970s and 1980s enabled the use of antibodies, a fundamentally novel form of cancer therapy. First was the hybridoma system that allowed the production of mouse monoclonal antibodies targeting human antigens (3). The second was the ability to graft a human antibody constant region to a mouse antibody variable region to generate chimeric antibodies with better therapeutic efficacy and fewer adverse effects (4). In 1975, researchers at Stanford used the hybridoma system to develop mouse antibodies targeting human lymphomas (5). The initial antibodies targeted unique immunoglobulin chains (named anti-idiotype antibodies) expressed only by cancerous B cells, but not by normal B cells. The breakthrough arrived in 1982 when the team at Stanford tested the anti-idiotype antibody in a patient with poorly differentiated B cell cancer. The anti-idiotype antibody named 4D6 induced remarkable tumor regression and symptom resolution (6). Although anti-idiotype antibodies produced remission in lymphoma patients, a unique anti-idiotype antibody had to be created for each patient and such personalized targeting was not commercially viable. The team had to select an antigen that would be expressed in most patients with lymphomas and decided to target the pan-B cell antigen CD20. The research group subsequently developed an antibody targeting CD20, that negated the need for patient-specific be-spoke antibody development. Working with a then-fledgling biotech company IDEC, the team grafted the anti-CD20 antibody onto a human antibody constant region to develop the first chimeric CD20-targeting antibody IDEC-C2B8 (7), later renamed rituximab. In 1994, the first-in-human trials of rituximab demonstrated remarkable efficacy in B cell lymphomas (8, 9) leading to FDA approval in 1997 (**Figure 1B**). Two observations underline the significance of this CD20-targeting antibody approval. First, CD20 is now one of the most common targets in oncology with eight FDA-approved antibodies, ADCs, and BsAbs directed against the antigen (10) and several more in regulatory review by the FDA. Second, rituximab, which was acquired by Genentech (now Roche), produced more than 6 billion USD in annual global sales in 2019 (1). Rituximab approval marked a paradigm shift in cancer treatment, introducing the concept of targeted immunotherapy that avoided many of the toxicities associated with cytotoxic chemotherapy. Rituximab shifted the focus from solely cytotoxic approaches to precision medicine, changing how researchers approached drug development and opening the field of antibody-based drugs in oncology.

### Trastuzumab, the first successful antibody for solid cancer

Patients with solid tumors such as lung, breast, or colon cancers, far outnumber those with B cell cancers and other hematologic malignancies. While B cell cancers could be targeted by pan-B cell antigens such as CD20, such tumor-associated antigens were not identified in solid tumors. Most solid tumors are surrounded by a complex network of stomal cells which creates an immunosuppressive microenvironment and limited access to drugs, which leads to a far worse prognosis compared to hematologic malignancies. This immunosuppressive microenvironment and lack of tumor-specific antigens added to the difficulty of antibody-drug development in solid tumors. In 1987, pioneering studies at UCLA demonstrated that ~25% of patients with breast cancer overexpress the product of an oncogene named HER2 on the cell surface (11). Based on this groundbreaking discovery, scientists at Genentech used the hybridoma technique to generate a mouse monoclonal antibody, mumAb4D5 that targets the human HER2 antigen (12). In preparation for human clinical trials, the mumAb4D5 antibody was one of the first to undergo humanization (13) and was later named Trastuzumab. However, the path from antibody development to human trial was not straightforward. Genentech in the 1990s was a small biotech startup with little experience in developing cancer therapies and initially declined to proceed with the human trial. The decision was later reversed due to relentless advocacy by physicians and patient advocacy groups along with the urgings of some of Genentech’s scientists and executives (14). Human trials showed tumor regression in breast cancer patients that over-express HER2 (15), leading to the FDA approval of Trastuzumab in 1998 (**Figure 1**). Currently, several antibody drugs are available to target different HER2 epitopes and have shown dramatic efficacy in HER2-expressing breast and gastrointestinal cancers. In 2019, the Lasker Clinical Research Award recognized the development of the HER2-targeting antibodies in breast cancer (16). The same year, Genentech/Roche’s combined annual revenue from the sale of HER2 targeting antibodies exceeded 10 billion USD (1). HER2 is now the target of seven FDA-approved antibodies, ADCs and BsAbs, and more such HER2-targeting antibodies are expected to be approved in the coming years (10).

### Gemtuzumab ozogamicin, the first successful antibody-drug conjugate

Rituximab and trastuzumab utilized antibody-dependent cellular cytotoxicity (ADCC) or complement-dependent cytotoxicity (CDC) to kill cancer cells (17). While such antibodies provided precise targeting of specific cell types, ADCC and CDC were less potent at inducing cell death when compared to cytotoxic agents. Thus researchers focused on producing antibody-based drugs with higher sensitivity to cancer cell killing by combining the precision targeting offered by an antibody and the potent killing offered by cytotoxic drugs. This led to the development of the first ADC for the treatment of acute myeloid leukemia (AML). Compared to breast cancers or lymphomas, AML has a much lower incidence. But in the 1980s, patients with AML had a terrible prognosis particularly if they have relapsed disease or have advanced age. Two parallel events led to the creation of gemtuzumab ozogamicin. First was the studies conducted at the Fred Hutchinson Cancer Center that led to the generation of antibodies targeting CD33, an antigen expressed by the AML cells (18). The second event was the discovery of calicheamicin, a potent bacterial toxin isolated from a rock in Waco Texas by a vacationing scientist who worked for the Lederle laboratories division in the American Cyanamid (19, 20). Calicheamicin was too toxic for systemic administration in cancer patients. To increase the cancer-targeting specificity of calicheamicin, scientists at the American Cyanamid (later renamed Wyeth Laboratories) and Celltech (later acquired by UCB) created the ADC gemtuzumab ozogamicin by attaching calicheamicin to the anti-CD33 antibody (21, 22). Gemtuzumab ozogamicin is internalized upon binding to CD33, resulting in the release of the calicheamicin payload, which induces double-stranded DNA breaks leading to target cell death (20). Preclinical studies with gemtuzumab ozogamicin showed specific killing of CD33^+^ patient-derived AML cells allaying the toxicity concerns (22). This led to the first-in-human clinical trials of gemtuzumab ozogamicin in AML patients at the Fred Hutch (23). A significant percentage of relapsed AML patients obtained remission with Gemtuzumab ozogamicin (24) leading to accelerated FDA approval in 2000 (**Figure 1**). The story of gemtuzumab ozogamicin then took an unexpected turn. In 2009, preliminary analysis from a two-arm phase 3 trial failed to demonstrate survival benefits and showed possible increased toxicity in patients receiving gemtuzumab ozogamicin (25). By 2010, gemtuzumab ozogamicin was owned by Pfizer through the acquisition of Wyeth, and Pfizer voluntarily withdrew the ADC. However, gemtuzumab ozogamicin continued to be evaluated through clinical trials at lower fractionated doses (26, 27). Collective data from the additional trials re-established the safety and survival benefit leading to its FDA approval in 2017. Currently, gemtuzumab ozogamicin in combination with chemotherapy is considered the standard of care in newly diagnosed CD33^+^ AML patients. Gemtuzumab ozogamicin led the way for ADC approval and currently, 12 ADCs are approved for the treatment of hematologic and solid cancers with a 13^th^ ADC under FDA and EMA review. Global annual sales of the currently available ADCs are projected to exceed $16.4 billion in 2026 (28) making ADCs one of the most successful drug formats.

### Blinatumomab, the first successful bispecific antibody

Cancer-targeting antibodies bind the target antigen on cancer cells and induce cancer cell death by activating various immune cells, such as NK cells and macrophages through the Fc receptors. However, as T cells lack Fc receptors, antibodies cannot activate T cells and thereby fail to mobilize one of the most potent killers against the cancer cells. In 1982, researchers at the University of Texas described the T cell receptor (TCR) that allows T cells to recognize antigens displayed on target cells (29). This discovery enabled the redesigning of antibodies to allow binding to the TCR complex, and redirect T cells against target cells (30, 31). Such antibodies were named bispecific T-cell engagers. However, these bispecific T-cell-engaging antibodies fell behind conventional antibodies in clinical testing due to the difficulty in producing sufficient clinical-grade material. This changed in 2000 when a group at the Max Delbrück Center in Germany developed a bispecific antibody by fusing a CD19-targeting scFv for B cell binding with a CD3-targeting scFv (CD3 is part of the TCR-CD3 complex that activates T cells) for T cell binding (32). The T-cell engaging bispecific antibody named bscCD19 × CD3 (later named MT103, then AMG103, now blinatumomab) was produced in sufficient amounts using Chinese hamster ovary (CHO) cells, remained in the monomeric form under a range of conditions, and met the criteria for clinical trial testing (33). A series of *in vitro* studies showed blinatumomab can recruit T cells to efficiently kill various B cell malignancies and normal B cells at very low concentrations (34, 35). Micromet a German-American biotech company was enlisted to initiate human clinical trials. However, investigators moved cautiously as in the past, T-cell activating biologicals had a track record of producing severe dose-limiting cytokine storms that terminated their clinical development (36–38). Blinatumomab testing in mice or other common animal models was unfeasible, as it did not bind mouse CD19 or CD3 antigens. Blinatumomab was found to be cross-reactive to CD19 and CD3 antigens only in chimpanzees (39), the human’s closest living relative. To establish safety and pharmacokinetics, blinatumomab was tested in a few chimpanzees which showed adequate T cell activation, B cell depletion, and minimal adverse effects (39). Multiple human trials ensued and showed tumor regression in several B cell lymphomas (40) with an impressive performance in pre-B ALL (41). In 2012, Amgen acquired Micromet to gain access to blinatumomab (42) anticipating its FDA approval, which followed in 2014 (**Figure 1B**) (43). Following the path laid out by blinatumomab, twelve BsAbs gained FDA and EMA approval in the last 2 years outpacing the approvals of monoclonal antibodies (**Figure 1B**). This is because when compared to monoclonal antibodies, BsAbs exhibit enhanced cytotoxicity and can kill target cells that express a low number of target antigens (44). Importantly, the BsAb format provides an alternative to chimeric antigen receptor (CAR) T cell therapies, with less complex treatment logistics that enable wider adoption in developing nations.

Pharmaceutical companies frequently trademark distinctive T-cell engager formats, employing specialized architectures and methods to construct the antibodies. Notable formats include the bispecific T cell engager (BiTE) by Amgen, Duobody by Genmab, DART by MacroGenics, and Xmab by Xencor. Bispecific antibodies offer an off-the-shelf alternative to the autologous chimeric antigen receptor (CAR) T cell platform and often outcompete CAR T cells due to their ease of use, and the ability to be used in combination with chemotherapies and other anti-cancer agents. Global annual sales of the currently available BsAbs are projected to exceed $3.7 billion in 2027 (45) exceeding that of CAR T cells (46). Current bispecific antibody development does not involve testing in chimpanzees, an endangered species.

### Ipilimumab, the first checkpoint inhibitor

Until now, cancer-targeting antibodies induce tumor regression by directly binding to cancer cells causing cancer cell death. But the holy grail of cancer treatment was to find a way to activate a patient’s immune system against the cancer cells. In the 1980s, researchers in France discovered a T cell-surface protein named CTLA-4 (47, 48). Initially, CTLA-4 was thought to be a T cell activating receptor due to its similarity to CD28, the well-known T cell co-stimulatory receptor. Subsequent work led by some of the investigators at the University of Texas that years earlier discovered the TCR (29), correctly identified CTLA-4 as a negative regulator of T cell activation (49). In the 1990s, a series of studies from the same investigators (they moved to UC Berkeley) demonstrated that CTLA-4 blocking antibody activates T cells leading to remarkable tumor regression in several mouse models of solid tumors (50). The biopharmaceutical company Medarex acquired the license to develop CTLA-4 targeting antibodies from UC Berkeley. To prepare for a clinical trial, Medarex generated a fully human anti-CTLA-4 IgG1 antibody named MDX-010 (later renamed ipilimumab) (51, 52). At that time, there was a lot of hesitancy from pharmaceutical companies to initiate immunotherapy trials as prior immunomodulatory agents failed to improve survival in cancer patients (53). Relentless persuasion from the research scientists (including the group at UC Berkley that described CTLA-4 function and subsequently relocated to MSKCC to be near the pharmaceutical companies and clinical trial sites) finally led to the initiation of human trials. During the early phases of the trial, there was widespread skepticism about the potential of ipilimumab to provide meaningful benefit in cancer, including a report by CBS News predicting the failure of ipilimumab and Medarex (54). This skepticism was also driven by the discontinuation of the phase 3 clinical trial evaluating another CTLA-4 targeting antibody tremelimumab by Pfizer in 2008, based on interim data that suggested a lack of benefit (55). But things changed in 2009 when a significant number of patients with melanoma experienced long remissions with ipilimumab. Encouraged by the preliminary clinical trial results, BMS acquired Meradex to obtain full ownership of ipilimumab (56). The final phase 3 trial data were released in 2010 and showed an overall survival benefit in melanoma patients (57). Ipilimumab received FDA approval in 2011 (**Figure 1B**). In the face of these remarkable results, the (former) reporter from CBS News had to acknowledge the benefit of ipilimimab (58). Tremelimumab, whose development was discontinued by Pfizer in 2008 based on interim results, also demonstrated survival benefit with longer follow-up (59). Tremelimumab (now acquired by AstraZeneca) is now FDA-approved for the treatment of multiple solid tumors.

Medarex also developed Nivolumab, an antibody targeting a second T cell inhibitory molecule PD1, discovered by a group at the Kyoto University (60). The acquisition of Medarex provided BMS with the ownership of nivolumab, which received FDA approval in 2014. Currently, more than 10 checkpoint-blocking antibodies have received FDA approval and they are considered standard of care in a wide range of cancers including melanoma, non-small cell lung cancer, renal cell cancer, hepatocellular carcinoma, Hodgkin lymphoma, and breast cancer (17). In 2018, the Nobel Prize was awarded to recognize the groundbreaking work on CTLA-4 and PD-1 pathways that established the field of cancer immunotherapy. In 2019, the combined annual sale of ipilimumab and nivolumab was close to 10 billion USD and accounted for ~75% of revenue generated by BMS.

### Pembrolizumab, the first tissue-agnostic drug approval

Before 2017, the FDA approved cancer drugs based on the drug’s activity on specific cancerous tissue types such as melanoma or non-small cell lung cancer. That changed with the approval of pembrolizumab based on the presence of a biomarker irrespective of the cancer-tissue type (tissue agnostic). Pembrolizumab development started at a small branch of the biotech company Organon, which was based in Cambridge MA. In the 1990s, following the discovery of PD1 as a negative regulator of T cells (60), scientists at Organon aimed to develop PD1 agonist antibodies that can suppress T cell function (61). The goal was to use such PD1 agonist antibodies to turn off runaway T cell activation in patients with autoimmune disorders. But the antibody program only yielded strong PD1 antagonists, that augmented T-cell activity. Organon scientists were keenly interested in pursuing PD1 agonist antibodies in oncology. However, after a series of acquisitions, Organon eventually ended up under Merck’s ownership, where the PD1 program was deprioritized due to the then prevailing pessimism with immunotherapy. The successful ipilimumab trial in 2010 (57) by BMS changed everything. Merck urgently reactivated the PD1 program with the lead anti-PD1 antibody MK-3475 (later renamed pembrolizumab) in 2010 to compete with BMS. Around the same time, nivolumab (anti-PD1 antibody by BMS) was being tested at Johns Hopkins in several cancers including colon cancer. But in contrast to other solid tumors such as non-small cell lung cancer, nivolumab had a low response rate in colon cancer with only 1 out of 33 colon cancer patients responding to nivolumab (62). Researchers at Johns Hopkins speculated that the responding patient likely harbored defects in DNA repair mechanisms (mismatch repair deficiency tumor). Such defects lead to a 10-100-fold increase in mutations compared to tumors in patients with intact DNA repair (mismatch proficient tumor) causing tumor cells to display numerous mutated antigens which are recognized and killed by the T cells. However, this theory required evidence in the form of a clinical trial testing of anti-PD1 antibodies in mismatch-deficient tumors. After several unsuccessful attempts to convince pharmaceutical companies to support such a trial, funding was provided by Johns Hopkins. Merck was looking to catch up with BMS and provided the anti-PD1 antibody, pembrolizumab. The study enrolled patients with mismatch repair deficiency across 12 different cancer types and showed that more than half of these patients had a durable response to pembrolizumab (63, 64). The astonishing results led to the FDA approval of pembrolizumab in 2017 (**Figure 1B**) for any solid tumor with mismatch repair deficiency. These studies along with other bio-marker-driven trials greatly expanded the indications of pembrolizumab over its competitor nivolumab. Pembrolizumab is predicted to generate global sales of around 31 billion USD in 2025, the highest revenue generator across all drug categories (65).

Similar tissue-agnostic approvals are expected to increase in the coming years. In 2024, trastuzumab deruxtecan, a HER2-targeting ADC, received FDA approval for any solid tumors that express high levels of HER2 as determined by immunohistochemistry (IHC 3+). This approval was based on clinical benefit demonstrated by subgroup analysis of HER2 IHC 3+ tumors in patients with lung, colon, endometrial, cervical, ovarian, bladder, biliary tract, and pancreatic cancers (66–68). Antibodies targeting novel antigens such as B7-H3 and TROP2 are now being explored in multiple solid tumors and may expand the number of tissue agnostic approvals (17).

## Lessons from past antibody approval

The antibody-drug discoveries of the past reveal several important lessons. First, most antibody drugs originated from a partnership between academic labs and small biotechs (rituximab developed by IDEC, blinatumomab by Micromet, ipilimumab and nivolumab by Medarex, pembrolizumab by Organon, etc.). Large pharmaceutical companies play a critical role by conducting the major phase 3 trials required for the final regulatory approvals. Thus it is essential to maintain a close collaboration between academia and the biotech industry for the continued development of innovative antibodies, a view shared by experts in the field (69, 70). Second, each antibody drug required a core group of researchers who pushed forward despite early failures and apathy from the wider drug development community. The blockbuster antibody drugs were made possible due to the unrelenting labor of groups at Stanford, UCLA, Fred Hutch, University of Texas, Max Delbrück Center, MSKCC, Johns Hopkins and multiple other institutions. Third, antibody drug development is a convoluted process with multiple paths to success. Sometimes the target discovery preceded the antibody generation (CTLA-4 and ipilimumab, HER2 and trastuzumab, CD20 and rituximab). Occasionally the antibody was generated well before the optimal method of targeting could be discovered (mismatch repair deficiency targeting with pembrolizumab). And other times, the target-antigen and antibody were both available, but the optimal antibody-drug format took years to develop (CD33 targeting with gemtuzumab ozogamicin required the discovery of the drug calicheamicin, CD19 targeting with blinatumomab required optimization of the bispecific antibody platform). Fourth, drug approval is not a one-way street and approvals can be reversed with the failure of confirmatory trials. Gemtuzumab ozogamicin remains the only ADC that regained approval after an initial withdrawal and underscores the need for judicious trial design. In 2022, belantamab mafodotin an ADC initially approved for myeloma patients was withdrawn after the ADC failed to show survival benefit in a confirmatory trial (71). Subsequent clinical trials in 2024 with belantamab mafodotin demonstrated survival benefit (72). Belantamab mafodotin is now under FDA and EMA review and may repeat the events that played out with gemtuzumab ozogamicin a decade ago. Antibody development in oncology both influences and is influenced by advancements in other medical fields, particularly rheumatology. CD20 and CD19 targeting therapies, including antibodies and CAR T cells, were originally designed for the treatment of B-cell malignancies but are now inducing remissions in refractory autoimmune diseases such as systemic lupus erythematosus and rheumatoid arthritits (73–76). Similarly, pembrolizumab, the PD-1 blocking antibody with one of the broadest ranges of indications in oncology, was initially developed to suppress self-reactive T cells in autoimmune conditions like rheumatoid arthritis (61). This cross-disciplinary innovation continues today, with selective targeting of clonal T-cell receptors, initially conceptualized for the treatment of T-cell malignancies (77, 78), now being effectively applied to patients with clonal T-cell-mediated autoimmune disorders, such as ankylosing spondylitis (79).

Our attempt at highlighting the pivotal case studies in antibody development is imperfect. The list leaves out multiple groups that contributed substantially to shaping antibody research and development. Such groups include those who developed the phage display technology, the generation of transgenic mice that produced human antibodies, single B cell cloning for antibody discovery, and those working on Fc effector function characterization including Fc engineering to alter antibody half-life and increase killing functions, and many more. A glance at the list also shows that it is dominated by researchers working mostly at universities and biotechnology companies located in the United States. The sheer numbers provide some insight into the bias as a substantial fraction of scientists working in drug discovery along with the biotechnology or pharmaceutical companies were based in the United States (70, 80).

## Six events that may further shape antibody development

The remarkable success of antibody-based therapies in oncology has intensified research efforts toward generating additional formats with improved properties to overcome the limitations of the current approach. Some promising approaches that may again revolutionize antibody therapies include the following;

### Novel checkpoint inhibitors

Following the initial success of targeting CTLA4, PD1/PDL1 checkpoints, a range of additional immune checkpoints, such as LAG3, PVRIG, NKG2A, CD47, TIGIT, VISTA, TIM3 and OX40, underwent clinical trials in various cancers (17). Out of the multiple novel checkpoints, only LAG3 targeting by relatlimab demonstrated benefit in phase 3 clinical trial for patients with melanoma, leading to FDA approval (81). The underwhelming performance of the other novel immune checkpoint inhibitors has led the pharmaceutical industry to refocus on the PD1–PDL1 pathway, with 10 antibodies receiving FDA approval at the end of 2024 (**Figure 1B**). However, the new PD1/PDL1 targeting antibodies are not expected to offer a therapeutic benefit above that of the existing antibodies. This may change with ivonescimab, a novel bispecific antibody developed by Summit Therapeutics simultaneously targets PD1 and vascular endothelial growth factor (VEGF). A recent phase 3 data demonstrated that ivonescimab possesses superior activity in patients with non-small cell lung cancers when compared to the conventional PD1 targeting antibody pembrolizumab (82). Thus this format of dual targeting of PD1 and VEGF may revolutionize the development of checkpoint inhibiting antibodies in multiple tumor types.

### Novel antibody formats

The vast majority of antibody therapies utilize the IgG1 and IgG4 isotypes that allow bivalent binding to target antigens (17). The IgM isotype is a pentameric molecule with ten antigen binding sites thereby increasing the antigen binding avidity over a similar IgG molecule. An IgM-based antibody developed by IGM Biosciences showed promising results in the phase 1 trial and may provide an alternative to the widely used IgG format (83). Another attractive format is nanobodies that are obtained from the heavy-chain antibody in camelids. Nanobodies offer some distinct advantages over conventional IgG antibodies. Nanobodies require a simple manufacturing process and have excellent stability across a range of thermal and chemical conditions (84). This suggests that targeting cancer antigens with nanobody format may improve efficacy over the existing antibody-based formats with several such nanobodies being tested in early-phase trials (85). Finally, antibody-mimetics are a diverse group of organic compounds, composed of short chains of peptides or nucleic acid residues, and can specifically bind target antigens. Antibody-mimetics also offer several advantages over conventional antibodies including improved solubility, stability, and low production costs. The common antibody mimetics that have shown encouraging activity in oncology clinical trials include Affibodies (86), Darpins (87), and Aptamers (88).

### Multi-specific antibodies

After the exponential rise of bispecific antibodies in hematologic and more recently in solid tumors (89), multispecific antibodies may represent the next leap in this design approach. Multispecific can simultaneously engage more than two targets. This allows the engagement of two different cancer-associated antigens (for example, CD19 and CD20 in B-cell malignancies) along with engaging a cytotoxic T cell (by binding CD3) using the third binding domain (90). This approach prevents therapy resistance due to the loss of a single antigen (such as CD19 or CD20 loss) and the cancer cells have to down-regulate both to escape killing. Other multispecific antibodies engage two T cell receptors, such as CD3 and CD28 to deliver a more potent activating signal against the cancer cell (91). Alternatively, the antibodies can engage CD3 and CD8 to selectively activate the CD8^+^ T cells resulting in potent target cell killing while avoiding adverse effects such as cytokine storm (92).

### TCR mimic antibodies

Conventional antibodies cannot pass through the cell membrane, so they can only target antigens found on the cell surface. However, intracellular antigens can be displayed on the cell surface bound to MHC molecules, making them accessible to antibody targeting. Traditionally, such peptide-bound MHCs are recognized by TCRs and not by naturally occurring antibodies. Using phage display, researchers at Immunocore developed antibodies that can recognize peptide-MHC complexes such as gp100, which is overexpressed in melanoma and presented on the cell surface alongside HLA-A*02:01 (93). Antibodies are being developed to target other cancer-specific overexpressed intracellular proteins, such as human telomerase reverse transcriptase (hTERT), MUC1, NY-ESO1, TCR gamma alternate reading frame protein (TARP), p53, WT1, and preferentially expressed antigen of melanoma (PRAME) (94), with early clinical trial results for PRAME showing promising outcomes. Similarly, preclinical studies demonstrated that TCR mimic antibodies can target oncogenic mutations that are only present in cancer cells (95, 96). Thus TCR-mimic antibodies targeting intracellular antigens are expected to open up a range of new targets in different malignancies.

### Novel ADCs

Innovations in ADCs are driven by improvements in the linker technology or by the attachment of novel payloads to the antibody (97, 98). Linkers connect the cytotoxic payload to the antibody. The mc-VC-PABC linker developed by Seagen in 2003 (99) was widely adopted by multiple FDA-approved ADCs (17). Recently, Daiichi Sankyo developed a tetrapeptide linker (GGFG) that enables the attachment of a large number of hydrophobic payloads to the antibody while maintaining antibody stability and the desired pharmacokinetics (100). This tetrapeptide linker led to the generation of the HER2-targeting ADC trastuzumab deruxtecan, which showed superior efficacy over the conventional HER2-targeting ADC trastuzumab emtansine in a phase 3 trial (101). Such new linkers are now being adopted to improve drug attachment for multiple other ADCs (102). The ADC payloads have similarly evolved as well. The popularity of ADC payloads shifted from calicheamicin in the 1990s, to auristatins (MMAE and MMAF) around 2000 (99), and now with camptothecins starting around 2015 (98, 100). While auristatins and camptothecins remain the two most common payloads, newer agents such as protein degraders, Toll-like receptor (TLR) agonists and stimulator of interferon genes (STING) agonists are making their way into clinical trials (97).

### Alternate delivery methods

Most antibodies are delivered by intravenous infusions which is a time-intensive process, requires vascular access, and administration is limited to clinical settings. Increasingly, antibodies are being delivered by the subcutaneous route, thereby reducing delivery time, the need for intravenous access, and allowing antibody therapies outside of the physician’s office (103). Thus BsAbs and checkpoint inhibitors are rapidly adopting subcutaneous delivery systems to improve the convenience of treatment (104, 105). A recent phase 3 trial demonstrated survival benefits and reduced adverse effects with the use of subcutaneous delivery of antibodies in patients with epidermal growth factor receptor (EGFR)–mutated advanced NSCLC (106), indicating that the subcutaneous delivery may also offer a therapeutic benefit. The next step in facilitating the delivery of antibodies may involve oral antibodies. Delivery of antibodies by oral route is now being explored with the use of autoinjectors (107) or with nanoparticles (108). Although clinical trials have yet to commence evaluating the oral delivery of antibodies, this approach holds significant potential to streamline antibody therapies, rendering their administration as straightforward as that of small molecules.

We are in an age of synthetic biology, where genetic engineering allows the creation of antibodies and antibody-like molecules with desirable binding affinities, defined biochemical properties, and novel formats that do not exist in the natural world. We expect that synthetic biology will continue to reshape the landscape of antibody development, offering innovative formats to target a range of malignancies with unprecedented precision and effectiveness.

## Data Availability

The original contributions presented in the study are included in the article/supplementary material. Further inquiries can be directed to the corresponding authors.
